# Obituary

**Published:** 1878-02

**Authors:** 


### Obituary.

Another link between the present of Chicago and its earliest days, was severed by the death of WILLIAM BUTTERFIELD, on the 13th of January last, in the 57th year of his life. He was the last surviving son of Hon. Justin Butterfield, one of the pioneers of Chicago, and the leader in his day of its bar.

Mr. Butterfield's career is of interest to the medical profession in Chicago, inasmuch as he was the first graduate of Rush Medical College. Those who were present at the Commencment exercises of this institution, on February 15, 1876, will recall the fact that his name was mentioned by President, then Professor, J. Adams Allen, in the delivery of his historical address upon the early days of the college. The first regular organization of the school was effected in the fall of 1843, Drs. Brainard, Blaney, McLean and Knapp constituting the first Faculty. The lectures were delivered in little rooms on Clark street, opposite the Sherman House, where twenty students were in attendance, of whom one only was graduated, after a course of sixteen weeks' instruction. This graduate was Dr. William Butterfield.

He engaged in the practice of his profession for a few years after his studies were completed, but subsequently entered the regular service as First Lieutenant in the Marine Corps; and served as such during the Mexican war. While on duty in Mexico, his health became so impaired from the insalubrity of the climate that he remained ever afterwards an invalid. In the late civil war he served as Brigade Commissary of Subsistence till the close of the contest. Since then he has lived in the retirement of private life.

He was a man of more than ordinary strength of mind and fortitude of character, displaying those qualities in an eminent degree during his last lingering and distressing illness; and he closed a life of unobtrusive patriotism and Christian piety cheered by the affectionate solicitude and attentions of a numerous family, who can point with pride to their father's career.

